# Regeneration of Rabbit Calvarial Defects with Combination of Stem Cells and Enamel Matrix Derivative: A Microcomputed Tomography and Histological Evaluation Comparing Two- and Three-Dimensional Cell Constructs

**DOI:** 10.3390/medicina60030451

**Published:** 2024-03-08

**Authors:** Kyung-Hwan Na, Hyun-Jin Lee, Ji-Eun Lee, Jun-Beom Park

**Affiliations:** 1 Department of Medicine, Graduate School, The Catholic University of Korea, Seoul 06591, Republic of Korea; nabali8@gmail.com; 2 Department of Periodontics, College of Medicine, The Catholic University of Korea, Seoul 06591, Republic of Korea; hyunjinlee0423@gmail.com; 3 Department of Periodontics, Korea University Guro Hospital, Seoul 08308, Republic of Korea; 4 Dental Implantology, Graduate School of Clinical Dental Science, The Catholic University of Korea, Seoul 06591, Republic of Korea

**Keywords:** cell differentiation, cell survival, estradiol, osteogenesis, stem cells

## Abstract

*Background and Objectives*: This study addresses the challenge of bone regeneration in calvarial defects, exploring the efficacy of stem cell-based therapies and enamel matrix derivative (EMD) in tissue engineering. It assesses the regenerative potential of two- and three-dimensional cell constructs combined with mesenchymal stem cells (MSCs) and EMD in rabbit calvarial defects. *Materials and Methods*: This research involved the use of bone-marrow-derived MSCs cultured in silicon elastomer-based concave microwells to form spheroids. White rabbits were grouped for different treatments, with Group 1 as control, Group 2 receiving only EMD, Group 3 getting EMD plus stem cells, and Group 4 being treated with EMD plus stem cell spheroids. Computed tomography (CT) and microcomputed tomography (micro-CT) imaging were used for structural assessment, while histological evaluations were conducted using hematoxylin and eosin, Masson’s trichrome, and Picro-sirius red staining. *Results*: CT and micro-CT analyses revealed varying degrees of bone regeneration among the groups. Group 4, treated with three-dimensional MSC spheroids and EMD, showed the most significant improvement in bone regeneration. Histological analyses corroborated these findings, with Group 4 displaying enhanced bone formation and better collagen fiber organization. *Conclusions*: The study supported the biocompatibility and potential efficacy of three-dimensional MSC constructs combined with EMD in bone regeneration. Further investigations are needed to confirm these findings and optimize treatment protocols.

## 1. Introduction

Bone defects caused by inflammation, trauma, tumor removal, or congenital anomalies pose a serious obstacle to bone regeneration, prompting the exploration of tissue engineering solutions [1]. Extensive research has been conducted on bone grafting materials to enhance bone healing and regeneration [2,3]. A variety of bone grafting materials, including autografts, allografts, xenografts, and synthetic graft substitutes, are being utilized to promote bone formation and provide a framework for new bone formation [2]. Autogenous bone is considered the gold standard for bone defect reconstruction, but it has limitations such as donor site morbidity, longer surgery times, and limited supply [4]. Additionally, graft materials can cause inflammation and interfere with effective bone regeneration, requiring a more efficient and minimally invasive treatment approach [5,6,7].

In the field of dentistry, bone grafting techniques are widely applied, including alveolar ridge augmentation for dental implants and reconstructive procedures for significant bone defects [7]. Nevertheless, achieving optimal bone regeneration remains a complex endeavor. To address these issues, stem-cell-based therapies are emerging as an alternative in the field of bone tissue engineering [8]. Mesenchymal stem cells (MSCs) are well known for their self-renewal, multilineage differentiation, and immunomodulatory properties [9]. The use of MSCs with their multipotent differentiation potential shows promise for regenerative medicine [10]. Enamel matrix derivative (EMD) has been used frequently in periodontal regeneration procedures [11]. It contains amelogenin, which stimulates osteoblast activity and promotes bone formation [12]. Combining MSCs and EMDs in a tissue engineering approach may have the potential to improve bone regeneration outcomes [13]. While traditional two-dimensional cell cultures have been widely used in research, recent advances in tissue engineering have highlighted the advantages of three-dimensional cell structures [14]. Compared to two-dimensional cultures, three-dimensional cell structures better mimic the in vivo environment, improving cell interactions, extracellular matrix synthesis, and tissue organization [15]. This approach enhances cell viability, proliferation, and differentiation, making it an attractive option for tissue regeneration.

This study aims to evaluate and compare the regenerative potential of two- and three-dimensional cell constructs combined with MSCs and EMD for calvarial defect repair in rabbits. Computed tomographic scans, microcomputed tomography (micro-CT), and histological analyses will be employed to assess the effectiveness of these interventions in promoting bone regeneration, tissue morphology, and osteogenic differentiation.

## 2. Materials and Methods

### 2.1. Manufacturing Stem cell Spheroids and Designing Animal Model and Groups

Mesenchymal stem cells derived from bone marrow at passage 5 were utilized for this study [16]. Then, the stem cells were then seeded at a density of 1 × 10^6^ cells per well onto silicon elastomer-based concave microwells (StemFIT 3D; MicroFIT, Seongnam-si, Gyeonggi-do, Republic of Korea) and cultivated in the media.

Four male New Zealand white rabbits, each with a weight of 3.5 kg, were subjected to a rigorous selection process. These rabbits were certified as specific pathogen-free (SPF), ensuring a controlled and pathogen-free environment throughout the duration of the study. The selection procedure, animal care practices, and surgical protocols and preparations adhered strictly to the approved guidelines established by the Institutional Animal Care and Use Committee (IACUC) at the College of Medicine, The Catholic University of Korea, Songeui Campus. All surgical interventions, as well as pre-surgical and post-surgical animal care, were provided in accordance with the Animal Protection Act, the Laboratory Animal Act, the Guide for the Care and Use of Laboratory Animals, and the Guidelines and Policies for Rodent Survival Surgery, as specified by the IACUC at the College of Medicine, The Catholic University of Korea (Approval number: CUMS-2022-0209-03).

### 2.2. Surgical Protocol

**Figure 1:**
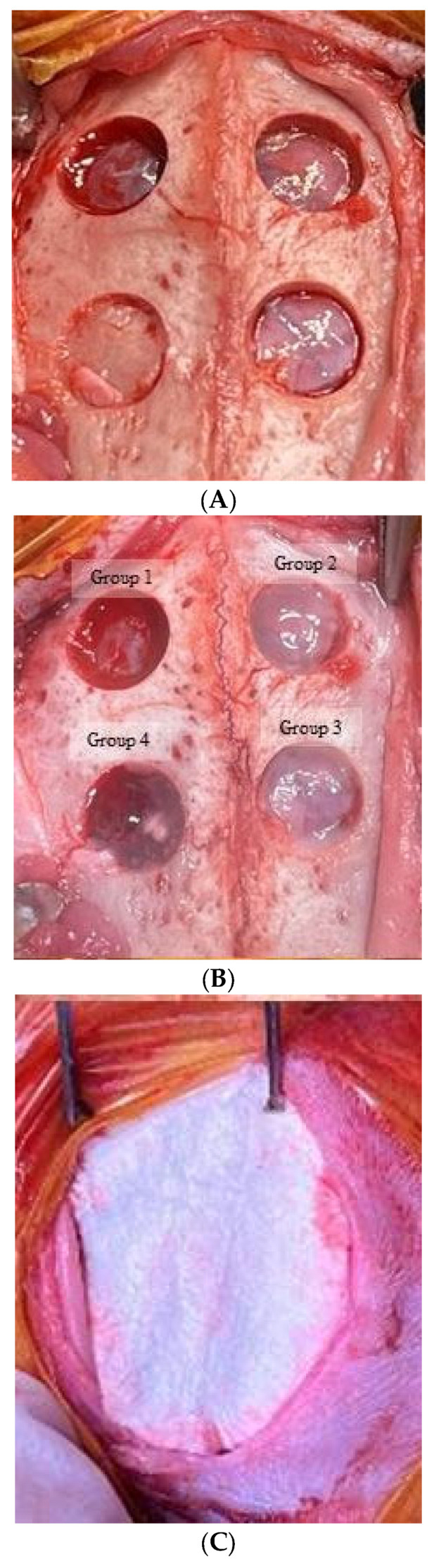

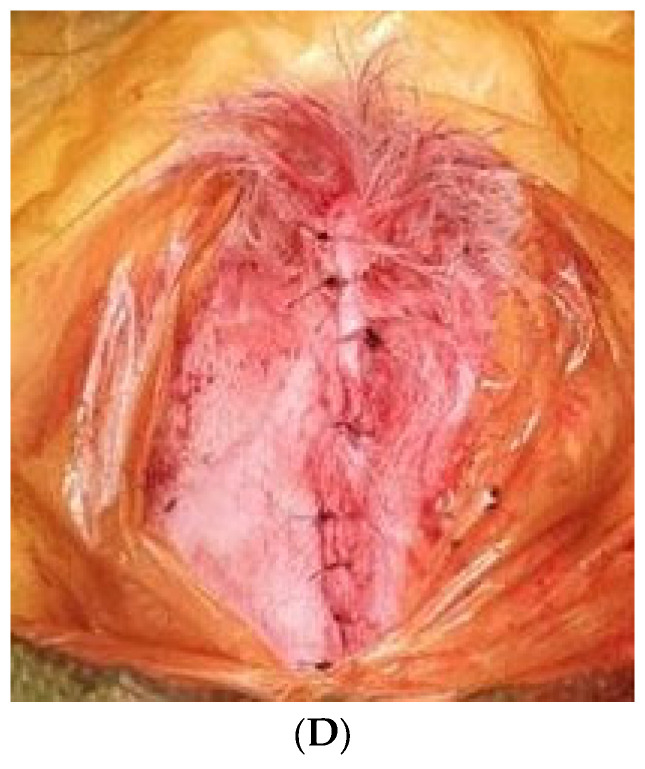
The surgical procedures employed in the study. (**A**) The procedure commenced with the exposure of the bone, followed by the creation of four uniform circular defects, each with a diameter of 6 mm, using a trephine bur. (**B**) These defects were then filled with different experimental materials, corresponding to the designated groups. The group assignments were as follows: Group 1 served as the control group, where defects were left untreated to naturally undergo the healing process without any intervention; Group 2 received 0.07 mL of enamel matrix derivative (EMD) only onto the defect site; Group 3 was treated with EMD in combination with 1.0 × 10^6^ stem cells; and Group 4 utilized EMD along with stem cell spheroids. These spheroids represent clusters of stem cells cultivated via a three-dimensional cell culture technique. (**C**) Post-application of the experimental materials, each defect was covered with a collagen membrane. (**D**) Finally, the surgical flaps were reapproximated and secured using absorbable sutures.

General anesthesia was administered to all subjects through intravenous injections of ketoprofen (3 mg/kg) and ketamine (10 mg/kg). The cranial region of the rabbits was prepared by shaving and disinfecting it with povidone iodine. A midline incision was carefully made from the frontal bone to the occipital bone, and a full-thickness flap was subsequently raised. Following bone exposure, four uniform, circular defects with a diameter of 6 mm were generated using a trephine bur (Figure 1A). This specific defect size was chosen based on the previous reports [9,17,18]. The defects were filled with different experimental materials based on the group they were assigned to, with Group 1 as control, Group 2 receiving only EMD (0.07 mL; Emdogain^®^, Straumann, Basel, Switzerland), Group 3 receiving EMD plus stem cells, and Group 4 being treated with EMD plus stem cell spheroids (Figure 1B). The defects were subsequently covered with a collagen membrane (Collagen membrane 2, Genoss, Suwon, Korea) (Figure 1C). The flaps were repositioned and closed using absorbable suture material (5-0 Vicryl^®^, Ethicon, Somerville, NJ, USA) (Figure 1D).

### 2.3. Reconstruction of Computed Tomography Image and Measurements

Computed tomography (CT) scans on the rabbits were performed at 2-, 4-, 6-, and 8-week intervals using the CT scanner (Canon Aquilion Lightning 160 Model TSX-036A, Canon Medical Systems, Tochigi, Japan), equipped with Software v 8.4. The CT scanning parameters remained consistent throughout the study, including a tube voltage of 120 kV, an mA setting of 18, and a precise slice thickness of 0.5 mm. This approach enabled the sequential imaging of the rabbit specimens, allowing for the observation of structural changes at different time points. These sequential scans provided a comprehensive assessment of the evolving changes within the calvarial regions of the specimens over the designated time intervals [19].

To analyze the collected data from these sequential scans, VR 3D imaging software (Vitrea v8.1, Canon Medical Systems Corporation, Otawara-shi, Tochigi, Japan) was utilized. This software facilitated a detailed examination of any temporal variations in radiographic densities within the 5 mm bone defect regions among the various experimental groups. Additionally, 3D reconstruction techniques were applied at each interval to gain a comprehensive understanding of the evolving anatomical parameters within the calvarial defect area, offering valuable insights into the dynamic changes occurring over time as a result of the experimental interventions. Hounsfield Units within predetermined regions of interest across the calvarial defects served as the basis for quantifying bone mass, utilizing automated and standardized calculations to maintain consistency. The imaging software’s calibrated tools facilitated precise quantification of the sagittal and transverse dimensions of the defects, enabling accurate assessments of distance and area. This methodology was essential for tracking the progression of bone healing and the closure of defects. Designated points within the CT images were used for longitudinal monitoring, allowing for the evaluation of changes at intervals of 2, 4, 6, and 8 weeks, thereby providing a comprehensive temporal analysis of the healing process.

### 2.4. Microcomputed Tomography Analysis

At 8 weeks post-implantation, the animals were euthanized, and calvarial bone samples were promptly collected and fixed in 4% (*w*/*v*) paraformaldehyde for subsequent micro-CT analysis. Prior to micro-CT analysis, the specimens designated for measurement were positioned within the micro-CT scanner (SkyScan1173; Bruker-CT, Kontich, Belgium) with the center appropriately aligned. The measurement process utilized SkyScan1173 control software (Ver 1.6, Bruker-CT), employing a tube voltage of 130 kVp, a tube current of 60 μA, one mm aluminum filtration (filter), an exposure time of 500 ms, a pixel resolution of 2240 × 2240 pixels, and a pixel size of 13.93 μm. The scanner was rotated at intervals of 0.3 degrees through a full 180-degree rotation, resulting in a collection of 800 high-resolution images. Subsequently, for the cross-sectional reconstruction, an image with 2240 × 2240 pixels was generated using Nrecon software (Ver 1.7.0.4, Bruker-CT), and alignment of the cross-sectional images was accomplished using Dataviewer (Ver.1.5.1.2, Bruker-CT). Data analysis was conducted by defining the region of interest using CTAn software (Ver 1.17.7.2, Bruker-CT), and quantification of the volume of new bone formation within the specified area was carried out by applying a threshold range of 39–255 to identify and assess the bone content and parameters within the region. Microcomputed tomography (micro CT) analysis was conducted to quantify the bone volume (mm^3^), percent bone volume (%), and bone mineral density (g/cm^3^).

### 2.5. Immunohistochemical Staining Process and Histological Assessment

Sections of the surgical sites were initially fixed in 10% formalin for a period of 10 days. Following this fixation period, the specimens underwent a series of procedures including decalcification and trimming, eventually being embedded in paraffin. Decalcification pre-treatment involved the use of a commercial decalcification reagent (CalciClear Rapid; National Diagnostics, Atlanta, GA, USA) to remove mineral deposits. This meticulous decalcification process spanned two months and employed acid-based techniques. Following successful decalcification, the processed tissue was prepared for embedding in paraffin blocks, laying the foundation for further analysis. From the paraffin-embedded blocks, serial coronal sections with a thickness of 5 μm were prepared. The central section encompassed the whole defect area. An elaborate deparaffinization and hydration process was meticulously carried out to optimize tissue clarity and staining precision. This process involved a gradual transition of slides through varying concentrations of ethyl alcohol, from 100% to 70%.

Histological analysis, using hematoxylin and eosin staining, was conducted on rabbit calvarial bone blocks obtained from surgical sites two weeks after the surgical intervention. Histologic evaluation using hematoxylin and eosin staining is described below. Following this preparatory phase, the tissue sections were immersed in Mayer hematoxylin for a duration of 10 min, followed by a thorough rinse with tap water. This crucial step facilitated the visualization of cellular components and detailed structural analysis within the bone specimens. Further enhancement of tissue structure differentiation was achieved through contrast staining using a 1% alcoholic eosin Y solution. The staining process was concluded with precise graded alcohol washes, which meticulously included sequential immersions in 70%, 95%, and two steps of 100% ethanol to refine contrast and effectively remove excess stain. To preserve the integrity of the stained calvarial bone sections, coverslips were meticulously mounted using Permount™ Mounting Medium (Electron Microscopy Science Hatfield, PA, USA), ensuring secure fixation and long-term preservation.

For the implementation of Masson’s trichrome staining, a meticulously orchestrated sequence of steps was executed. Following this, a 5 min incubation in Weigert iron hematoxylin solution was performed to achieve robust nuclear staining. To remove excess hematoxylin, a thorough 10 min tap water rinse was administered. Introduction of contrast to cytoplasmic elements was achieved through a 3 min application of Biebrich scarlet solution. Subsequently, sections underwent a 2 min exposure to a 3% phosphomolybdic-phosphotungstic acid solution for effective differentiation. Aniline blue staining, lasting 5 min, imparted a distinctive blue hue to collagen fibers. Post-staining, sections were rinsed thoroughly in tap water and immersed in a 1% acetic acid solution for 1 min to optimize staining results. A final brief tap water rinse prepared the sections for subsequent dehydration. Ethanol dehydration ensued, meticulously transitioning through 70% ethanol, 95% ethanol, and culminating with two immersions in 100% ethanol.

The systematic application of Picro-sirius red staining involved a series of precise steps. Nuclei were first stained with Weigert’s hematoxylin for 5 min, followed by a thorough 10 min wash in running tap water. Subsequently, the sections underwent staining in Picro-sirius red for one hour, ensuring a state of near-equilibrium staining. To refine the staining, two changes of acidified water were employed for rinsing, with the removal of excess water achieved through shaking or, for select slides, blotting with damp filter paper. Dehydration was carried out meticulously through three changes of 100% ethanol. The slides were then cleared in xylene, providing transparency, and mounted for subsequent detailed analysis. This stringent staining protocol, designed for advanced research, aimed to provide a comprehensive understanding of the tissue microarchitecture under investigation. The systematic approach ensures precision and reliability in the obtained staining results for robust analysis.

These stained slides were then digitized using the scanner (Panoramic 240 Flash III scanner, 3DHISTECH Ltd., Budapest, Hungary) to create digital representations of the stained tissue sections for comprehensive analysis and documentation.

### 2.6. Statistical Analysis

The data were presented as means and standard deviations of the experiment. Normality and equality of variance were assessed. Group differences were analyzed using Kruskal–Wallis test (SPSS 12 for Windows, SPSS Inc., Chicago, IL, USA); test was used to assess group differences (SPSS 12 for Windows, SPSS Inc., Chicago, IL, USA; *p* < 0.05).

## 3. Results

### 3.1. Computed Tomographic Evaluation

**Figure 2:**
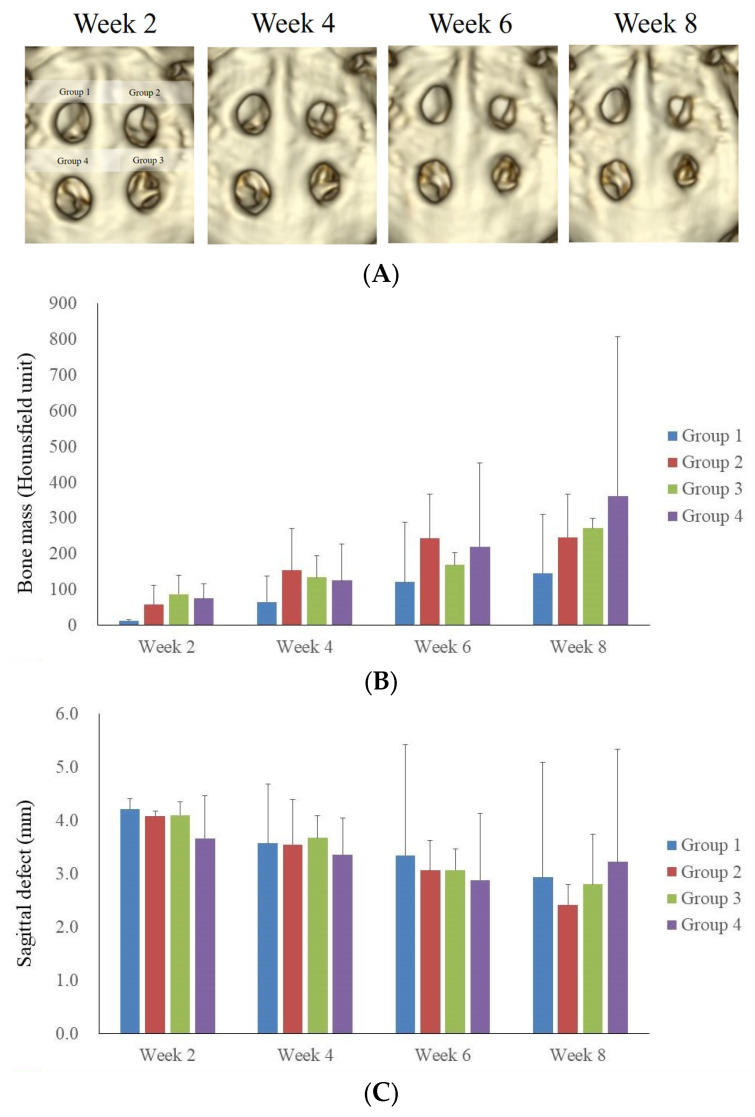

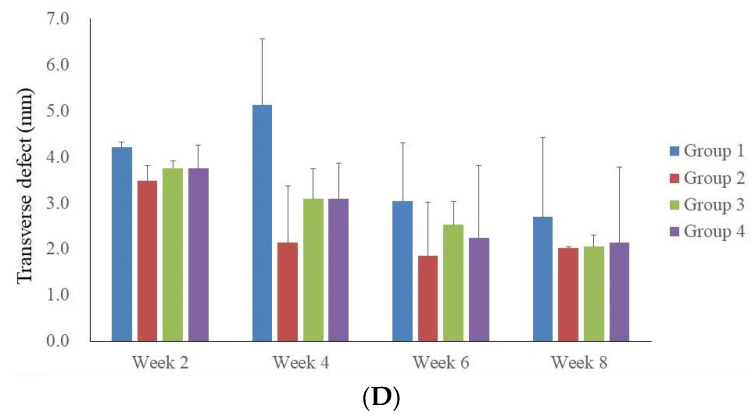
Computed tomography (CT) evaluation of calvarial defect. (**A**) This panel presents a time-lapse series of CT images, capturing the progression of calvarial defect healing over a period of 2 to 8 weeks. Sequential scans depict the healing process of four calvarial defects, illustrating an early phase of postoperative radiolucency to healing of bony defects. (**B**) The changes of Hounsfield units are used to quantify bone healing over eight weeks in four experimental groups. Initial low HU values indicate post-operative bone loss. By the fourth week, a modest increase in HU suggests the onset of bone regeneration. Notable variations between the groups were noted by week 6. By week 8, Group 4 shows the highest HU, suggesting a more effective healing process. (**C**) This part illustrates the longitudinal measurement of calvarial defect closure in rabbits, viewed in the sagittal plane. It highlights the average defect size at bi-weekly intervals, demonstrating a general trend of defect reduction. At 2 weeks, the defect sizes are similar across all groups. Weeks 4 and 6 showed a modest reduction in defect size, with variance among groups indicating different healing rates. By week 8, a trend towards defect closure is observed in all groups, although none achieve complete regeneration. (**D**) This segment evaluates the changes in calvarial defect closure in rabbits, employing a sagittal transverse defect methodology. At the initial 2-week mark, the defects in all experimental groups were of comparable size, indicative of a uniform early postoperative status. Measurements taken at 4 and 6 weeks post-surgery showed a reduction in defect size across the groups, albeit with minor variations between them, suggesting different rates of healing. By the 8-week interval, a general trend towards defect closure was observed in all groups.

Figure 2A presents a time-lapse progression of the healing of calvarial defects over a period of 2 to 8 weeks, as visualized through CT imaging. This figure comprises sequential CT scans depicting the healing process of four distinct calvarial defects. Initially, the early postoperative phase, marked by radiolucency, is evident at 2 weeks, particularly in Group 1, where an observation of high-density and bone-like structures was noted. This early sign of bone formation can be attributed to the body’s natural bone healing phases, initiated by an inflammatory response leading to a reparative phase. This phase is followed by a gradual healing by the fourth week, indicating the onset of bone healing. By the sixth week, there was more advancement towards decrease in the size of the defect. The observations recorded at the 8-week mark indicated a markedly enhanced healing process in Group 4. This group demonstrated the most significant progression in terms of bone regeneration and defect closure compared to the other groups studied.

Figure 2B demonstrated the progression of Hounsfield units in the context of bone mass over an eight-week period, across four groups. The measurement of Hounsfield units served as a quantitative indicator of bone density changes during the healing process. Immediately following surgery, all groups exhibited low Hounsfield unit readings, indicative of initial post-operative result. A gradual increase in Hounsfield unit values by the fourth week suggests the commencement of bone regeneration. Notably, by the sixth week, there was observable variability in HU values among the different groups. By the eighth week, Group 4 exhibits the highest Hounsfield unit readings, suggesting a more advanced healing outcome in comparison to the other groups.

Figure 2C illustrated the longitudinal measurement of calvarial defect closure in rabbits, using a sagittal view, over an 8-week period. The figure quantifies the closure of sagittal defects, in millimeters, across four experimental rabbit groups. A bar graph was employed to represent the mean defect size at bi-weekly intervals, highlighting a consistent trend of gradual defect diminishment. Initially, at week 2, the defect sizes are comparable among all groups, without any statistically significant differences in closure rates. Progressing to weeks 4 and 6, a modest reduction in defect size is observed, albeit with noticeable variations within each group, indicating differential healing trajectories. By the conclusion of the 8-week period, although all groups exhibit a continued trend towards defect closure, the extent of closure varies, and none of the groups achieve complete regeneration.

Figure 2D depicted the changes in calvarial defect closure in rabbits, measured using CT in the transverse plane over an 8-week period. At the 2-week interval, the defects across all groups are similar in size, indicating a similar postoperative condition. Subsequent measurements at weeks 4 and 6 demonstrate a decrease in defect size, with a slight inter-group variation suggestive of differential healing rates. By week 8, although there is a continued trend toward closure in all groups, the data showed variability in the extent of healing, and none of the groups have achieved complete regeneration of the calvarial defect.

### 3.2. Microcomputed Tomographic Measurement

**Figure 3:**
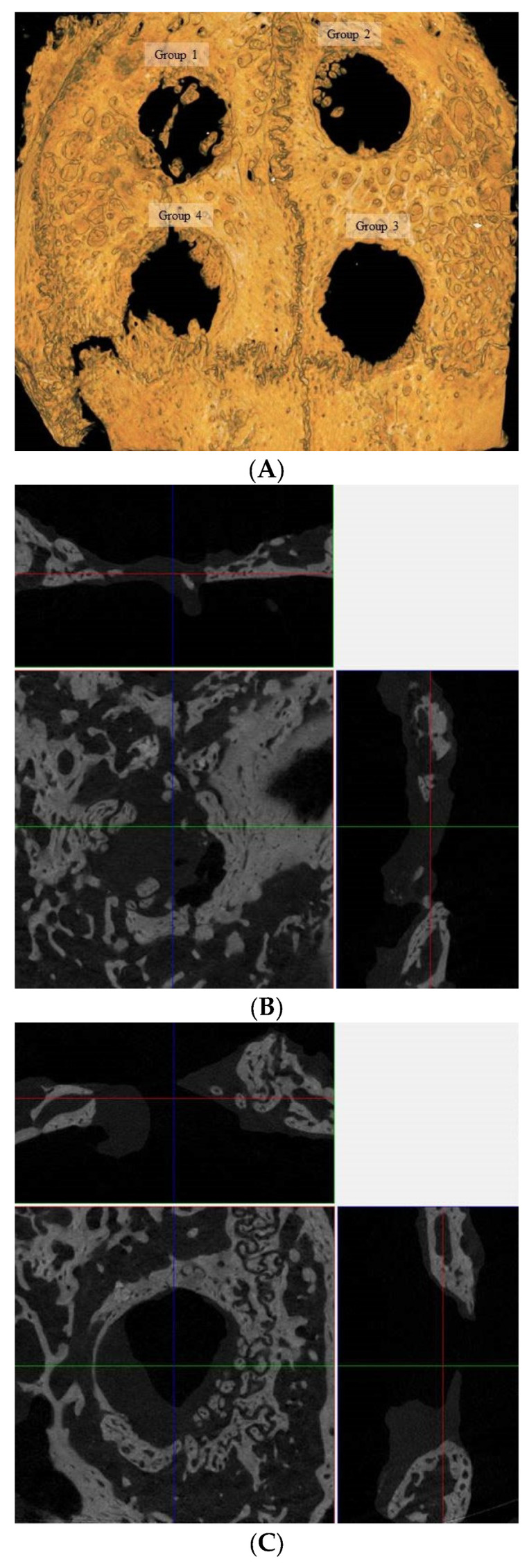

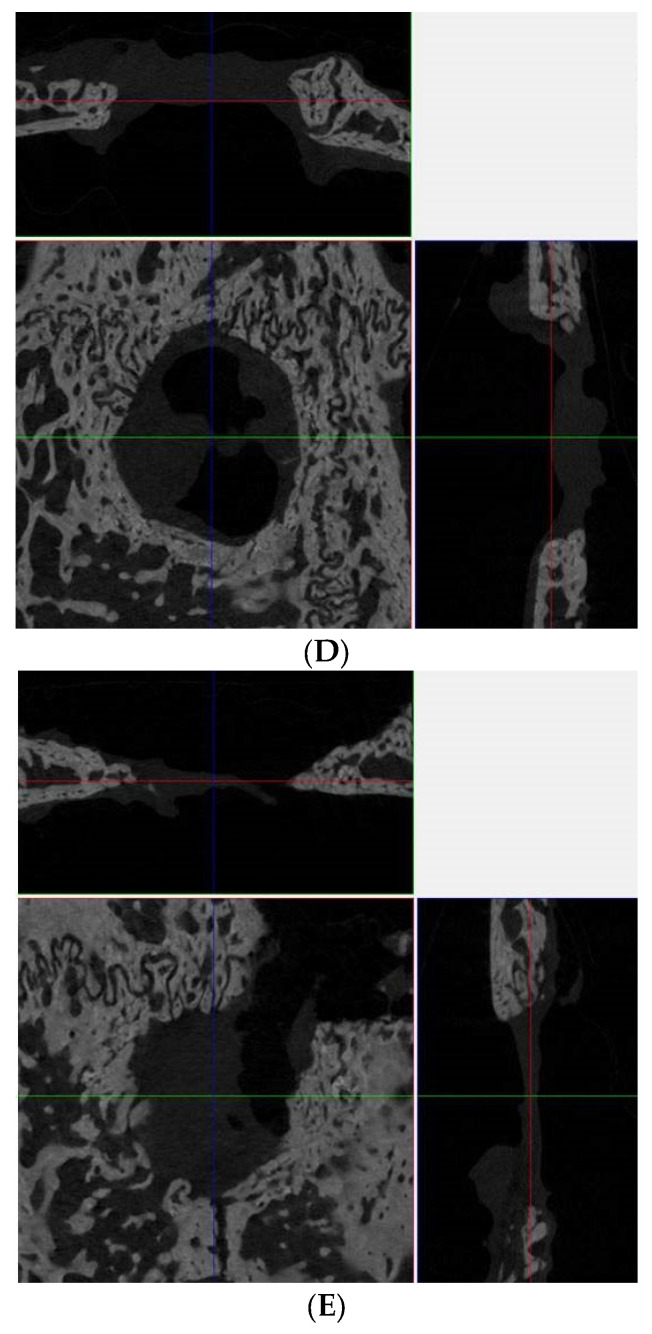
Microcomputed tomographic evaluation. (**A**) This section presented the evaluation of bone regeneration in rabbit calvarial defects across four groups. Group 1 exhibited a largely unfilled defect, indicative of limited bone regeneration. In contrast, Groups 2 and 3 demonstrated progressive improvements in bone healing, though these were similar to each other and showed a modest enhancement compared to the control. Group 4 exhibited the most pronounced bone regeneration within the calvarial defects, characterized by increased density and a more complete bone structure at the defect site. Furthermore, the margins of the defect in Group 4 were less distinguishable, suggesting new bone growth that was more seamlessly integrated with the adjacent bone tissue. (**B**) A multidimensional evaluation of calvarial bone defect regeneration at 8 weeks post-treatment for Group 1 was conducted from superficial, coronal, and sagittal views. (**C**) Multidimensional views are provided for Group 2, depicting the state of calvarial bone defect regeneration 8 weeks post-treatment. (**D**) The evaluation for Group 3 encompasses superficial, coronal, and sagittal views of calvarial bone defect regeneration, also at the 8-week post-treatment mark. (**E**) For Group 4, the figure showed superficial, coronal, and sagittal views of the calvarial bone defect regeneration 8 weeks following treatment. The colored lines represent sections of the coronal and sagittal views.

This study investigated calvarial defect regeneration in rabbits 8 weeks following intervention, encompassing four experimental groups: Group A served as the control with no therapeutic intervention, Group B received EMD as a standalone treatment, Group C was treated with a combination of two-dimensional cultured MSCs and EMD, and Group D was administered a combination of three-dimensional cultured spheroids and EMD (Figure 3A). The micro-CT images provided a high-resolution visualization of the bone structure and the extent of defect closure within each group. Group 1 showed a large, clearly defined defect with minimal evidence of new bone formation around the periphery. Group 2 exhibited a similarly sized defect with slight indications of new bone at the defect margins. In Group 3, there is a similar progression of bone growth into the defect area and the defect remains predominantly open. Group 4 presented with a reduced defect size, characterized by promoted new bone growth that nearly spans the entire defect area. Multidimensional evaluation including superficial, coronal, and sagittal views of calvarial bone defect regeneration at 8 weeks post-treatment from groups 1 through 4 were shown in Figure 3B–E, respectively.

**Figure 4:**
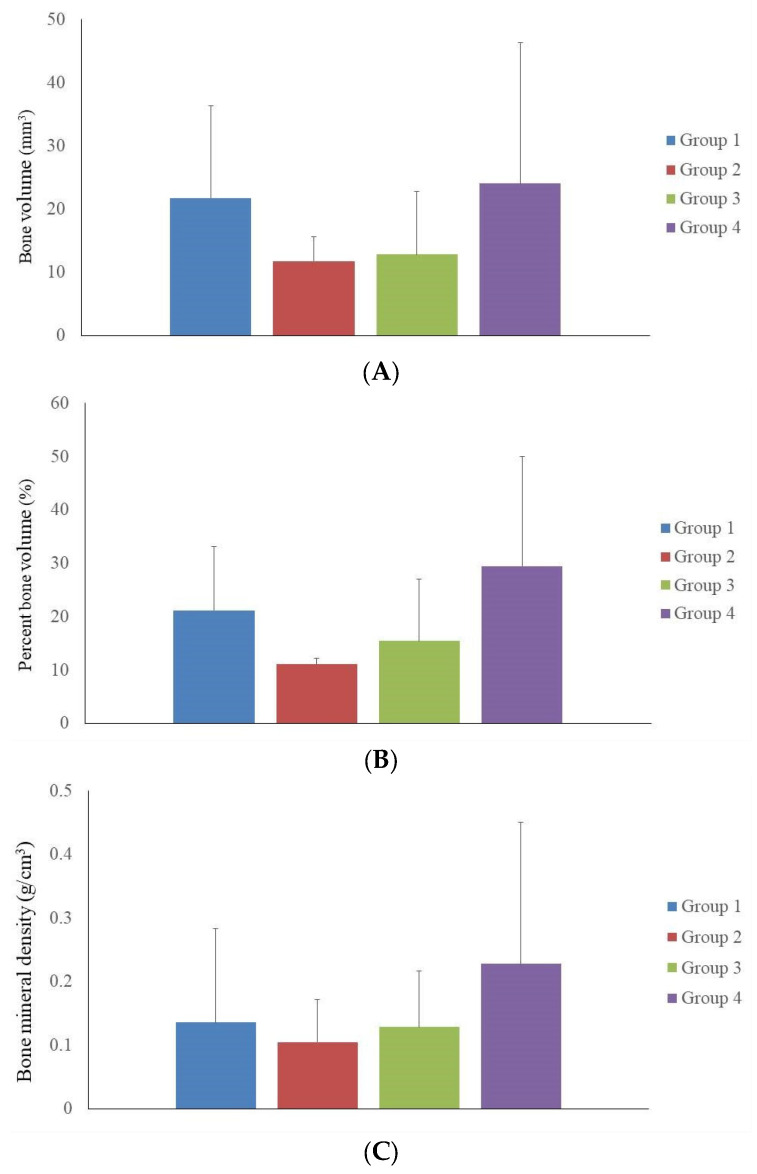
The quantitative evaluation of calvarial defects using microcomputed tomography. (**A**) The quantitative evaluation of bone volume from micro-CT analysis among four experimental groups at 8 weeks post-treatment. The bone volume assessment revealed that Group 4 exhibited the highest mean volume. (**B**) A comparative analysis of percent bone regeneration from micro-CT data. The graph displays the percentage of bone volume relative to the total volume of the calvarial defect. Group 4 again presented the highest mean percentage at 29.46 ± 20.54%, suggesting superior bone formation. (**C**) The bone mineral density measurements for the four study groups. The control group exhibited a baseline BMD of 0.14 ± 0.15 g/cm^3^. In comparison, Groups 2 and 3 showed BMD values of 0.10 ± 0.07 g/cm^3^ and 0.13 ± 0.09 g/cm^3^, respectively. Notably, Group 4 demonstrated the highest BMD at 0.23 ± 0.22 g/cm^3^, indicating a pronounced improvement and underscoring the potential effectiveness of the treatment strategies employed in this group.

Figure 4A presents the quantitative evaluation of bone volume from micro-CT analysis among four experimental groups at 8 weeks post-treatment. The bar graph quantifies bone volume. The control group (Group 1) established the value with a volume of 21.8 ± 14.5 mm^3^. Groups 2 and 3 showed modest increases in bone volume to 11.8 ± 3.8 mm^3^ and 12.8 ± 10.0 mm^3^, respectively. The bone volume assessment revealed that Group 4 exhibited the highest mean volume at 24.0 ± 22.3 mm^3^. However, the observed differences in bone volume compared to the control group did not reach statistical significance. Figure 4B illustrates a comparative analysis of percent bone regeneration from micro-CT data. The graph displays the percentage of bone volume relative to the total volume of the calvarial defect. The control group’s regeneration was at 21.13 ± 1.96%, with Groups 2 and 3 showing increments to 11.04 ± 1.12% and 15.44 ± 11.59%, respectively. In evaluating the percentage of bone volume, Group 4 again presented the highest mean percentage at 29.46 ± 20.54%, suggesting superior bone formation. Figure 4C demonstrates bone mineral density of four groups. The control group presented a foundational bone mineral density of 0.14 ± 0.15 g/cm^3^, with Groups 2 and 3 showing the results of 0.10 ± 0.07 g/cm^3^ and 0.13 ± 0.09 g/cm^3^, respectively. The assessment of bone mineral density further supported the efficacy of the treatments in Group 4, which showed the highest bone mineral density at 0.23 ± 0.22 g/cm^3^.

### 3.3. Histological Analysis with Hematoxylin and Eosin, Masson’s Trinchrome, and Picro-Sirius Red Staining

**Figure 5:**
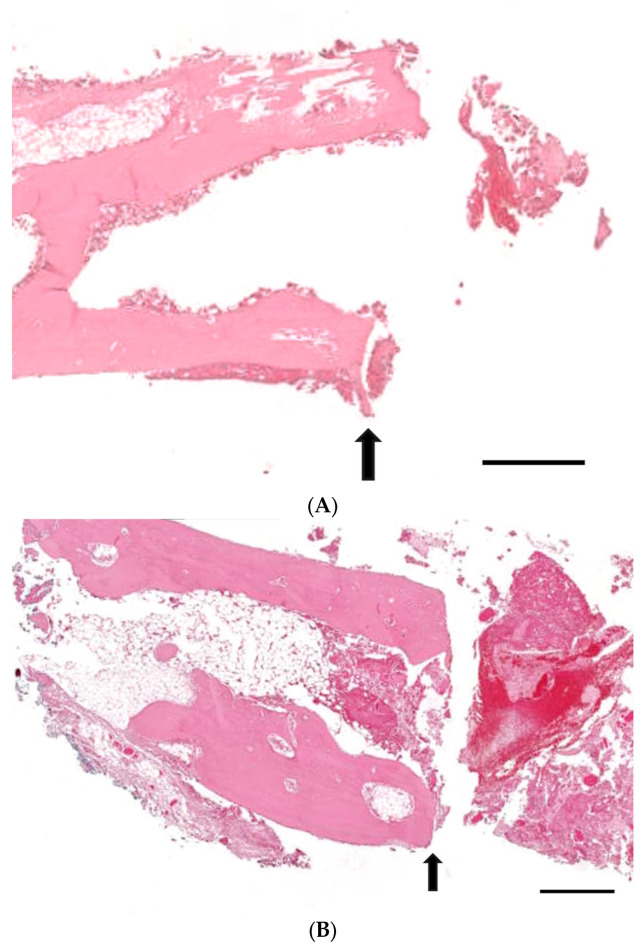

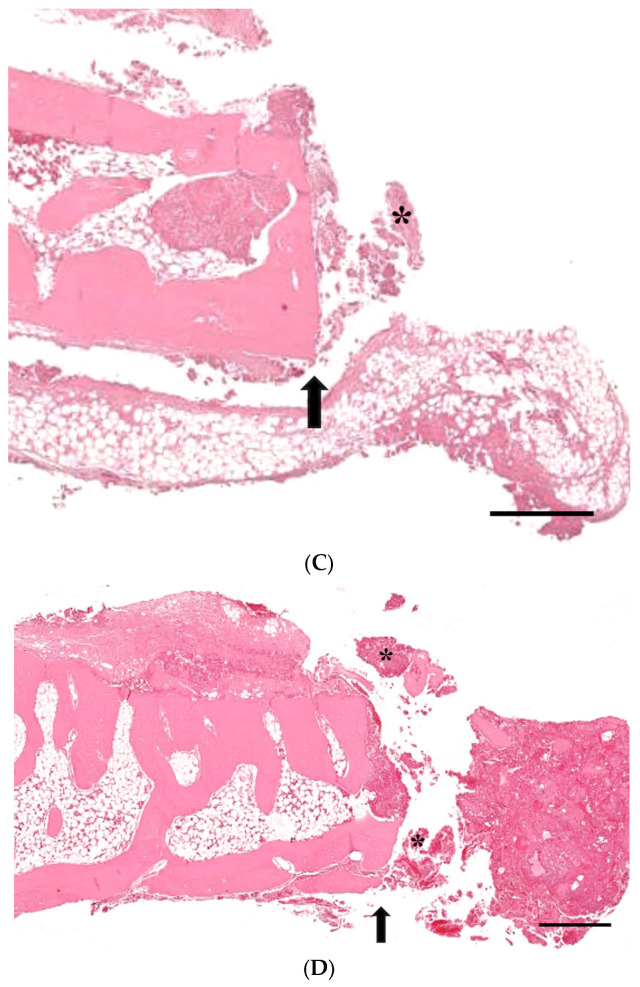
Histological evaluations of calvarial defects stained with hematoxylin and eosin in four experimental groups. Group 1 served as the control, Group 2 received enamel matrix derivative (EMD) alone, Group 3 was administered EMD combined with stem cells, and Group 4 was treated with EMD and stem cell spheroids. (**A**) Histological analysis of Group 1 revealed minimal new bone formation and a pronounced defect space. (**B**) In Group 2, there was a slight increase in new bone formation; however, the defect was largely unoccupied by new bone tissue. (**C**) Group 3’s sections indicated more substantial bone deposition compared to Groups 1 and 2, but the defect still exhibited incomplete bridging. (**D**) The specimens from Group 4 demonstrated a more significant bone formation, suggestive of a more robust healing response, although the defect had not achieved full regeneration. The defect margin is indicated by arrows, while asterisks (*) denote areas of new bone formation. The scale bar represents 500 μm.

Figure 5 presents histological sections of calvarial defects stained with hematoxylin and eosin across four experimental groups with Group 1 as control, Group 2 receiving only EMD, Group 3 getting EMD plus stem cells, and Group 4 being treated with EMD plus stem cell spheroids. The tissue response in Group 1 is characterized by the presence of minimal new bone formation and a large remaining defect space (Figure 5A). Group 2 showed slightly increased new bone formation, yet the defect remains substantially unfilled (Figure 5B). The histological outcome for Group 3 is described in Figure 5C, showing further increased bone deposition but still incomplete bridging of the defect. The response in Group 4 showed greater bone formation, indicating an enhanced healing response, although the defect is not fully regenerated (Figure 5D).

**Figure 6:**
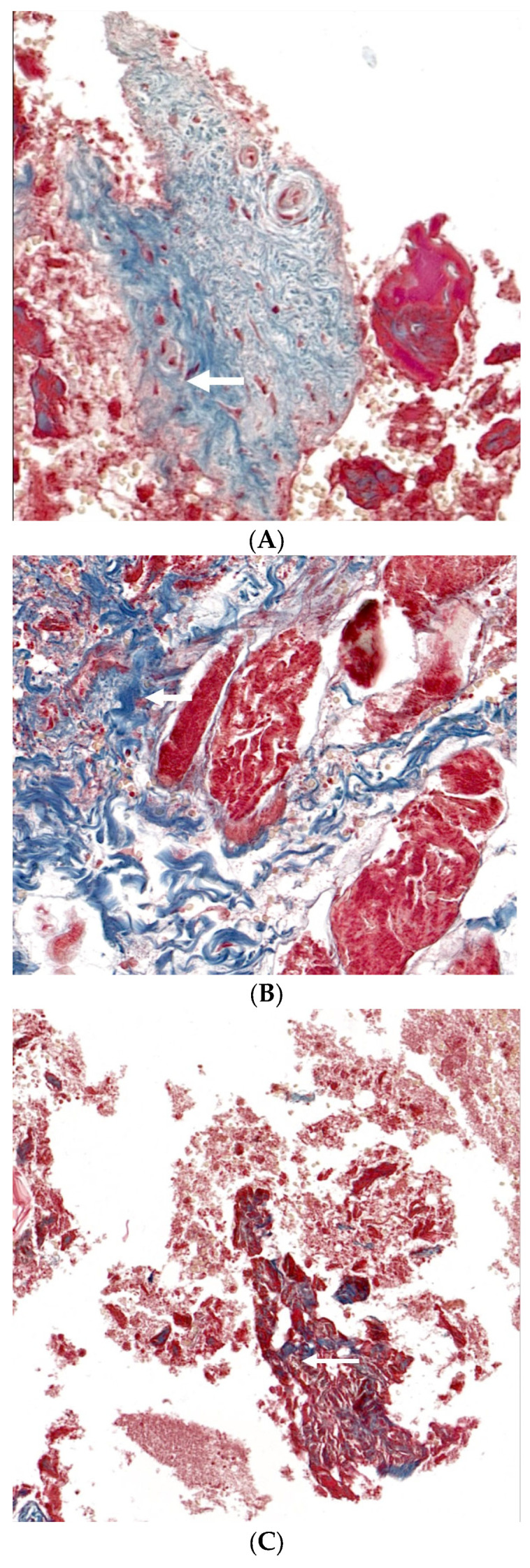

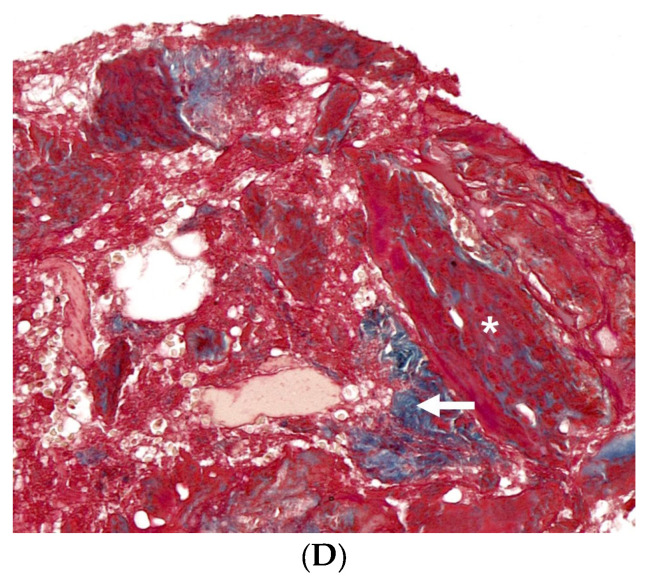
The histological evaluations of calvarial defects in four groups, utilizing Masson’s trichrome staining technique. The groups include Group 1 (control), Group 2 (treated with enamel matrix derivative (EMD) only), Group 3 (receiving EMD combined with stem cells), and Group 4 (treated with EMD and stem cell spheroids). Masson’s trichrome staining distinctively highlights collagen fibers and new bone formation, with collagen depicted in blue and new bone in red. (**A**) In Group 1, the histological sections showed minimal new bone formation, primarily indicated by sparse red staining, and a predominance of blue-stained collagen fibers, as indicated by arrows. (**B**) Group 2 demonstrated a marginal increase in red staining, suggesting enhanced new bone formation, though collagen continued to be the dominant component in the matrix. (**C**) The samples from Group 3 revealed increased areas of new bone formation within the collagen matrix, yet the defect was not completely bridged by new bone. (**D**) Group 4 exhibited significant new bone formation, as evidenced by extensive areas of red staining, indicating a more advanced stage of healing and defect closure. In the histological images, the presence of collagen is indicated by arrows, while asterisks (*) denote areas of new bone formation. The scale bar indicates 500 μm.

Figure 6 provides the histological evaluations of calvarial defects using Masson’s trichrome technique within four groups with Group 1 as control, Group 2 receiving only EMD, Group 3 getting EMD plus stem cells, and Group 4 being treated with EMD plus stem cell spheroids. This staining method differentially highlighted collagen fibers and new bone formation, with collagen depicted as blue and new bone as red. In Figure 6A, Group 1 exhibited minimal new bone formation, indicated by sparse red staining, and a predominance of blue-stained collagen fibers, as denoted by the arrows. Group 2 showed a slight increase in red staining, suggesting a greater presence of new bone, yet collagen remains the predominant matrix component (Figure 6B). Figure 6C illustrates the condition of Group 3, with increased areas of new bone formation amidst the collagen matrix, although the defect is not fully bridged by new bone. Group 4 displayed greater new bone formation, as evidenced by the substantial areas of red staining, suggesting an advanced stage of healing (Figure 6D).

**Figure 7:**
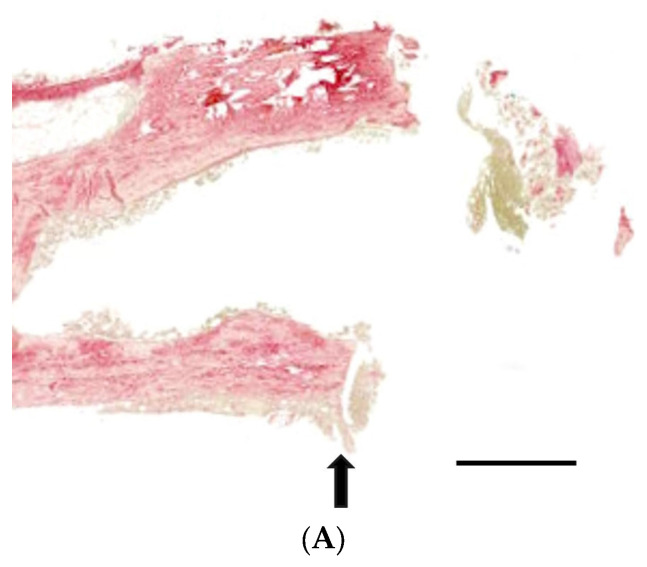

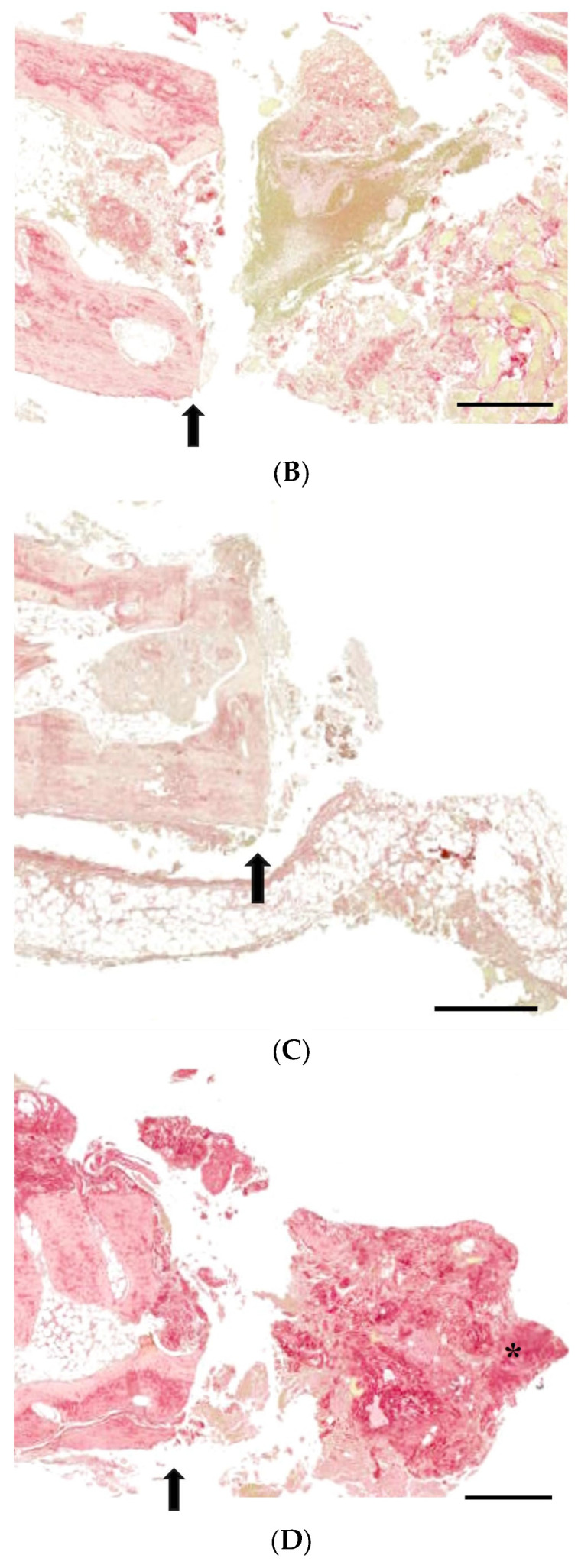
The histological assessments of calvarial defects in four experimental groups, utilizing Picro-sirius red staining. (**A**) In Group 1, the histological analysis revealed a diffuse and scant presence of collagen fibers, with limited evidence of organized tissue structure. (**B**) Group 2 exhibited a slightly increased density of collagen fibers; however, the overall tissue organization remained poorly defined. (**C**) The sections from Group 3 showed a more pronounced density in collagen deposition, with some regions indicating beginning tissue organization. (**D**) Group 4 displayed a notable increase in both the density and organization of collagen fibers, indicative of a more mature and structured healing response compared to the other groups. Arrows mark the boundaries of the defect, and asterisks (*) highlight regions containing collagen fibers. The scale bar represents a length of 500 μm.

Figure 7 exhibits histological sections of calvarial defects from four experimental groups, stained with Picro-sirius red. Figure 7A demonstrates the condition of Group 1, where there is a diffuse and scant presence of collagen without significant tissue organization. Group 2 showed a slightly denser accumulation of collagen fibers, though the overall organization remains poor (Figure 7B). As shown in Figure 7C, Group 3 demonstrated further density in collagen deposition with some areas. Group 4 revealed the increase in both the density and organization of collagen fibers, showing a more mature and structured healing response (Figure 7D).

## 4. Discussion

The aim of this study was to assess the regenerative capacity of two- and three-dimensional cell constructs in combination with MSCs and EMD for the treatment of bone defects. The results suggested the biocompatibility and functionality of three-dimensional construct of MSCs with EMD and its potential usefulness in the tissue engineering field.

Three-dimensional MSC constructs have been found to offer several advantages over conventional two-dimensional cultures [20]. The three-dimensional constructs are more effective in mimicking the natural tissue microenvironment, which can significantly enhance the differentiation potential of MSCs [14]. Research has indicated that 3D culture conditions can promote upregulated gene expression related to cell interaction proteins, such as β-catenin, integrin β1, and connexin 43. This environment also fosters the secretion of pro-regenerative cytokines, including vascular endothelial growth factor, hepatocyte growth factor, and interleukin-10, with a reported 2.1-fold increase in VEGF secretion, potentially contributing to improved tissue regeneration outcomes [21].

EMD is primarily composed of amelogenins, which are hydrophobic proteins representing over 90% of EMD’s total protein content [22]. EMD has been recognized as a bone graft substitute and has demonstrated efficacy in promoting regeneration in intrabony and Class II mandibular furcation periodontal defects [23]. Further, it has been reported that the integration of EMD into 3D cultures enhances osteogenic differentiation in MSC spheroids, evidenced by significant increases in Alizarin Red S staining intensity and RUNX2 mRNA expression levels, suggesting a potential role in bone tissue engineering applications [13]. Nonetheless, earlier research has indicated that the outcomes of combining guided tissue regeneration techniques with EMD could exhibit considerable variability, leading to unpredictable regenerative results [24].

The use of the membrane is based on its ability to function as a barrier designed to block soft tissue from penetrating into the defect site, promoting more targeted and effective bone regeneration [25]. This ability to maintain space was critical to establishing a controlled environment to evaluate the regenerative effects of the treatment being tested [26,27]. This membrane plays an important role in the management of periodontal defects, especially in the realm of guided tissue regeneration and guided bone regeneration methodologies [28]. In guided tissue regeneration applications, the membrane is used to treat defects within the periodontal ligament and alveolar bone [29]. They induce the growth of new periodontal tissue by blocking the rapidly proliferating gingival tissue from penetrating into the defect site, allowing the slower regenerating periodontal ligament and bone cells to occupy the area. This promotion is crucial for the regeneration of these structures. Similarly, guided bone regeneration uses membranes to induce bone regeneration in areas suffering from bone loss due to periodontal disease [30]. The membrane creates an environment where bone cells can migrate, proliferate, and mature, helping to restore lost alveolar bone. Since membranes may autonomously maintain the defect space, they are typically used in synergy with bone grafts [31]. A known limitation of collagen membranes is related to their suboptimal mechanical properties, which can cause them to collapse into the bone defect, so their use with bone grafts is recommended in clinical settings [32]. This study utilized a collagen membrane to impede the penetration of surrounding soft tissue, allowing osteogenic cells to repopulate the bone defect, while the bone graft reinforced the membrane to promote osteoblast proliferation [33]. These techniques are widely used in periodontics to address periodontal disease, which can lead to the loss of bone and tissue surrounding the teeth [34].

This study demonstrated that the integration of a three-dimensional construct of MSCs with EMD facilitated an enhanced regenerative response in calvarial defects. There was considerable variability as evidenced by the extended error bars in most groups. Moreover, the improvements made in the experimental approach of combining EMD with MSCs within a three-dimensional cell construct were marginal. A plausible explanation for this observation is the proportion of stem cells within the cultures may have been inadequate to significantly impact bone formation [35]. Fixing the stem cell construct utilizing a collagen membrane may not have provided adequate structural support. The application of a thermo-responsive or photo-responsive hydrogel, which exhibits reduced flow properties, could potentially enhance the stability and integrity of the cellular construct [36]. The study’s use of a specific time frame in its design may not be entirely suitable for generating significant differences in bone tissue development among the groups [37].

The determination of critical defect size for bone regeneration studies in rabbit calves is an important parameter for researchers working in the field of bone healing and tissue engineering [38,39]. This parameter is defined as the minimum defect diameter that does not spontaneously heal over the lifetime of the animal, which allows for the evaluation of different bone graft materials, growth factors, and tissue engineering strategies [40]. Earlier findings have identified a prevalent critical size defect of 15 mm (51.61%), typically situated in the central region (51.85%), with bilateral locations also noted (48.14%) [3]. The geometries of the defects were predominantly circular (66.66%), with rectangular (14.81%), square (14.81%), and ovoid (1.48%) shapes also reported [41]. Subsequent research indicated the use of a 10 mm defect in rabbit calvaria models [42], and another study identified an 8 mm defect as critical, located near the coronal suture on the parietal bone [43]. Additionally, a specific study demonstrated no significant difference in bone regeneration across a spectrum of defect sizes from 6 mm to 15 mm, challenging traditional notions of critical size [44]. Despite the benefits of employing critical size defects, the creation of four defects without mutual interference presents challenges and may lack reproducibility. Consequently, four uniform, circular defects, each with a 6 mm diameter, were created using a trephine bur, as supported by various previous studies [9,17,18].

Micro-CT can provide detailed three-dimensional evaluation of calvarial defects, providing crucial quantitative data on bone density, structure, and morphology, which is integral for monitoring treatment efficacy and understanding defect dynamics [45]. Its high-resolution, non-destructive imaging capabilities, and minimal sample preparation requirements render micro-CT an invaluable tool in both research and clinical diagnostics [46,47,48]. Micro-CT provided extremely high-resolution images of bone structures, allowing for detailed visualization of calvarial defects [46]. Micro-CT is characterized by its non-invasive nature, which enables the analysis of samples without necessitating any alteration or damage to the specimen being examined [47]. Compared to other imaging modalities that may necessitate extensive sample preparation, micro-CT requires reduced preparatory steps, rendering a more expedient and practical option for many analytical applications [46]. In one study, in vivo micro-CT was utilized to analyze bone remodeling in a rat model with calvarial defects, demonstrating the utility of micro-CT in tracking the dynamic process of bone remodeling over time, a critical factor in understanding the mechanisms of bone healing and regeneration [22]. Additionally, another investigation employed in vivo micro-CT to examine angiogenesis in rat calvarial flat bone defects, suggesting that this methodology was pivotal for assessing the formation and progression of new blood vessels during bone regeneration, showcasing micro-CT’s effectiveness in providing detailed insights into the physiological processes involved in bone healing [28].

Masson’s trichrome and Picro-sirius red staining are pivotal for the histological analysis of bone regeneration, allowing for the differentiation of tissue components. Masson’s trichrome staining is a significant and widely used technique in histological studies, especially in the differentiation and analysis of fibrosis in various tissues [49]. Moreover, Masson’s trichrome stain is used extensively in scientific studies of bone regeneration and plays a key role in understanding histomorphologic changes and the efficacy of various treatments [50]. In a previous study, Masson’s trichrome staining was used to evaluated the histomorphological changes during bone healing, demonstrating the utility of Masson’s trichrome staining in studying the cellular and molecular dynamics of bone regeneration [51]. In another report, Masson’s trichrome staining was utilized alongside hematoxylin and eosin staining and micro-CT [52]. In a study evaluating the impact of mesenchymal stem cell proliferation and differentiation on bone regeneration, Masson’s trichrome staining was used on tibial defects in an animal model to gain histological insight into the efficacy of stem cell-based therapies on bone repair and regeneration [53]. This study showed that combination of three-dimensional MSC construct and EMD displayed promoted new bone formation, as evidenced by the substantial areas of red staining, suggesting an advanced stage of healing and defect closure.

Picro-sirius red staining is a histochemical technique primarily used for the specific staining and visualization of collagen fibers within tissue samples [54]. Collagen plays a crucial role in the structural organization of tendons and other connective tissues [55]. It has become an essential method for studying collagen networks in various tissues, both under normal and pathological conditions [56]. This staining method is particularly useful in the examination of tissue remodeling and connective tissue pathologies. Picro-sirius red staining is also significant in clinical and research contexts for the quantitative assessment of collagen fibers. This quantification is particularly valuable in evaluating the response to therapeutic interventions or understanding the progression of diseases. Techniques like polarized light microscopy and circular polarizers are employed to enhance the visualization of collagen fibers stained with Picro-sirius red, facilitating both qualitative and quantitative analyses [57]. In this study, a combinatorial approach with stem cell construct and EMD led to an increase in both the density and organization of collagen fibers, indicative of a more mature and structured healing response.

This study found that the control group exhibited suboptimal outcomes, while the addition of EMD and MSCs in a three-dimensional construct led to moderately improved results. However, the evidence does not conclusively support the superiority of combining EMD with three-dimensional MSC constructs. Further research should employ well-characterized stem cell populations and extend the observation periods to comprehensively evaluate the efficacy of such interventions.

## 5. Conclusions

In summary, the results supported the biocompatibility of three-dimensional construct of MSCs with EMD and its potential usefulness as a bone graft material with moderately improved results. Future investigations with a more defined stem cell population and suitable recovery durations are warranted to elucidate the full potential of these regenerative strategies.

## Data Availability

This article contains all of the information that was created or examined during study.
